# Integrating machine learning, deep learning, and docking to predict aristolochic acid A carcinogenesis

**DOI:** 10.3389/fphar.2026.1873914

**Published:** 2026-07-20

**Authors:** Longzhu Li, Jiacheng Liao, Xintian Chen, Zeqiong Lin, Siqiao Gong, Junmin Huang, Ziqian Bi, Tianyang Wang, Xinliang Chia, Lu Chen, Yongzhi Xu, Huafeng Liu, Junfeng Hao, Jiansong Qi

**Affiliations:** 1 Guangdong Provincial Key Laboratory of Autophagy and Major Chronic Non-Communicable Diseases, Key Laboratory of Prevention and Management of Chronic Kidney Disease of Zhanjiang City, Affiliated Hospital of Guangdong Medical University, Zhanjiang, Guangdong, China; 2 Department of Gastroenterology, Affiliated Hospital of Guangdong Medical University, Zhanjiang, Guangdong, China; 3 National Clinical Key Specialty Construction Program (2023), Institute of Nephrology, Department of Nephrology, Affiliated Hospital of Guangdong Medical University, Zhanjiang, Guangdong, China; 4 Department of Computer and Information Technology, Purdue Polytechnic Institute, Purdue University, West Lafayette, IN, United States; 5 School of Advanced Technology, Xi’an Jiaotong-Liverpool University, Suzhou, Jiangsu, China; 6 JBT Technology Corp, Tainan City, Taiwan

**Keywords:** aristolochic acid A, bioinformatics, deep learning, machine learning, molecular docking, molecular dynamics simulation, renal cell carcinoma

## Abstract

**Objective:**

This study investigates the molecular mechanisms of renal clear cell carcinoma (RCC) induced by Aristolochic acid A (AAA) using machine learning, deep learning, and molecular docking approaches.

**Methods:**

To identify AAA target genes associated with RCC, differential expression analysis was performed on multiple datasets. Network toxicology, machine learning, deep learning, and molecular docking were used to explore the binding interactions between AAA and target proteins. The top candidate gene was validated using molecular dynamics simulation and *in vitro* Western blot assays.

**Results:**

A total of 74 genes were identified as potential targets in AAA-induced RCC. Subsequent machine learning analysis identified seven core genes as key regulators of RCC. Deep learning classification further highlighted five of these seven genes, including PYGL, ADH1B, PTGS1, EDNRA, and AURKA. Additionally, molecular docking simulations revealed strong binding affinities between AAA and these target proteins. Molecular dynamics simulation demonstrated the binding stability of the AAA-PYGL complex, and *in vitro* studies highlighted PYGL as a potential target of AAA. Elevated expression of PYGL was observed in both 786-O and AAA-induced HK-2 cells. Moreover, treatment with CP-91149 (a PYGL inhibitor) or PYGL knockdown restored the expression of E-cadherin, an epithelial-mesenchymal transition (EMT) marker, in HK-2 cells.

**Conclusion:**

By combining advanced computational methods with *in vitro* studies, this work elucidates a key toxicity mechanism of AAA in RCC. Our approach provides a feasible and efficient framework for toxicological studies, offering significant value for toxicologists with limited access to clinical specimens.

## Highlights

- Multidisciplinary Integration: Machine learning, deep learning, network toxicology, and molecular docking work synergistically to elucidate the molecular networks involved in Aristolochic Acid A-induced renal cell carcinoma.
- Core Gene Identification: Five core regulatory genes were identified that may play significant roles in Aristolochic Acid A-associated renal cell carcinoma. Among these core genes, PYGL, the top-ranked candidate, was validated through molecular dynamics simulation and *in vitro* study, highlighting the potential of these core targets as biomarkers and therapeutical targets.
- Translational Implications: This multidisciplinary approach bridges computational prediction and *in vitro* validation, enabling efficient elucidation of toxin-induced mechanisms and supporting safer evaluation of herbal compounds.

## 1 Introduction

Aristolochic acid (AA), predominantly found in plants of the *Aristolochia genus*, is linked to severe health risks. Earlier research showed a high incidence of upper urinary tract urothelial carcinoma, renal cell carcinoma (RCC), and liver cancer in Asian regions, including mainland China and Taiwan, which has been potentially linked to long-term AA exposure through medicinal and environmental pathways. In Europe, epidemiological studies have similarly linked AA exposure to urothelial tract cancers observed in patients with Balkan endemic nephropathy (BEN) (Slade et al., 2009). It was also discovered that tens of millions of individuals in Southeastern Europe, particularly in Romania, Serbia, and surrounding areas, may have been exposed to AA, which is associated with a high mutation burden that predominantly arises during the early clonal stages of tumor development (Lin et al., 2024; Senkin et al., 2024). In 2022, the International Agency for Research on Cancer (IARC) confirmed aristolochic acid A as a Group 1 human carcinogen, noting ongoing environmental and occupational exposure risks, and warned that further inaction will have far-reaching negative effects on the incidence of related cancers (Das et al., 2022).

Aristolochic acid is a mixture of compounds, including Aristolochic acid A (AAA), Aristolochic Acid B, and Aristolochic Acid C (Sborchia et al., 2019; Zhou et al., 2023), with AAA being the primary toxic component and indicator of AA concentration in medicinal materials (Sborchia et al., 2019). AAA is metabolized to Aristolactam nitrenium ions (AL), which bind to DNA bases and form AA-DNA adducts, which drive urinary system cancers (Zhang et al., 2024). Recent studies have shown that the induction of cytochrome P450 1A1 and P450 1A2 can inhibit AA-DNA adducts formation, offering a potential strategy to mitigate AA’s carcinogenicity (Stiborová et al., 2008; Dračínská et al., 2016; Komatsu et al., 2025).

Whole-genome and whole-exome sequencing analyses have elucidated the role of AA in TP53-related mutations and mutation signatures (e.g., SBS22), while emerging evidence links AAA-induced oxidative stress, apoptosis, fibrosis, and EMT to renal carcinogenesis. A recent study utilized the SBS mutational signature as a molecular fingerprint to define AA-related RCC, demonstrating that AA-induced tumor mutational burden reshapes the tumor immune microenvironment and confers a favorable response to immune checkpoint blockage (Lin et al., 2024). In this study, the historical prevalence of AA-containing herbal remedies in Taiwan was mentioned in the background context. However, patient stratification was not based on self-reported exposure history, a factor that is clinically challenging to rely upon for identifying AA-induced RCC cases. Despite this, no effective early biomarkers or therapeutic targets currently exist for AAA-associated renal cell carcinoma (RCC). In recent years, network toxicology, integrating multi-omics data, dynamic network modeling, and structure-activity predictions has emerged as a powerful approach to elucidate the complex interactions between toxicants and hosts (Chen et al., 2023; Liu C. et al., 2025). Given the urgency of this clinical challenge, our study presents an AI-based integrative framework that offers a rapid, cost-efficient strategy to identify potential molecular targets, thereby providing a foundational basis for future clinical studies on early diagnosis. Using AAA as a representative compound, we employed a multi-faceted systems toxicology framework to: (1) integrate multi-omics data to reconstruct the molecular network of AAA-induced RCC; (2) utilize topological and functional enrichment analyses in conjunction with machine and deep learning algorithms to identify key hub factors; (3) apply an integrated molecular docking strategy to validate the thermodynamic properties of the interactions between key targets and AAA; (4) assess the dynamic stability of AAA-PYGL complex by performing molecular dynamics simulation; (5) confirm findings through *in vitro* studies. This multidisciplinary approach bridged computational prediction and *in vitro* validation to efficiently investigate Aristolochic acid A toxicity mechanisms and accelerate evidence-based research.

## 2 Materials and methods

### 2.1 Acquisition of disease-related targets

Four publicly available renal cell carcinoma (RCC) transcriptomic datasets (GSE40435, GSE53757, GSE15641, and GSE68417) were curated from the NCBI GEO database. These four datasets represent general RCC samples and corresponding controls, in details GSE40435 (platform GPL10558, 101 adjacent non-tumour and 101 tumor samples), GSE53757 (platform GPL570, 72 normal and 72 tumor samples), GSE15641 (platform GPL96, 23 normal and 69 cancerous human kidney samples), and GSE68417 (platform GPL6244, 14 normal and 35 tumor samples). These four datasets contain a total of 487 samples, including 277 clear cell renal cell carcinoma (ccRCC) tumor samples and 210 control samples (adjacent non-tumor or normal kidney tissues). These samples were selected for their relevance to RCC pathogenesis and varying cohort sizes to enable robust model training and validation. Data preprocessing was performed using R (Version 4.4.3) with limma and GEOquery packages, including background correction, quantile normalization. GSE40435 and GSE53757 served as the discovery cohort, while the remaining two datasets (GSE15641, and GSE68417) comprised the validation cohort. To address batch effects, a multi-stage normalization pipeline was implemented. Surrogate Variable Analysis (SVA) was employed to model and adjust for latent confounding factors within the discovery cohort using the SVA package. Furthermore, ComBat Harmonization was applied to further correct for residual batch variations through parametric empirical Bayes frameworks. Following these corrections, Principal Component Analysis (PCA) demonstrated improved inter-batch sample clustering in reduced-dimensional space, confirming the success of data harmonization.

### 2.2 Acquisition of chemical components and targets of aristolochic acid A

Aristolochic Acid A (AAA) was characterized through multi-source database integration. Its physicochemical properties and biological parameters were systematically obtained from public databases, such as PubChem and ChEMBL, while canonical 2D structural descriptors (SMILES: COC1 = CC = CC2 = C3C(=C(C=C21)[N+](=O)[O-])C (=CC4 = C3OCO4)C (=O)O) were extracted from the PubChem database. Target prediction employed a tripartite strategy: ChEMBL Database: Ligand-receptor interaction profiling; SwissTargetPrediction: Chemical genomics-based prediction (http://www.swisstargetprediction.ch); PharmMapper: 3D pharmacophore matching (http://lilab-ecust.cn/pharmmapper). All predicted targets were restricted to the *Homo sapiens* proteome.

### 2.3 Differential gene expression analysis

Transcriptomic data were analyzed using the limma package. Differentially expressed genes (DEGs) were identified with thresholds of FDR-adjusted p < 0.05 and |log2FC| > 0.585 (1.5 foldchange). Results were visualized using ggplot2.

### 2.4 Weighted gene co-expression network analysis (WGCNA)

A scale-free co-expression network was constructed using the WGCNA package. Sample Quality Control (QC) involved outlier removal through hierarchical clustering. Soft Thresholding was performed using dynamic tree-cutting to determine the optimal power (scale-free R^2^ > 0.85). Module Detection included hierarchical clustering of topological overlap matrix (TOM) with minModuleSize = 30; Module-Trait Association assessed module eigengene-phenotype correlation (Pearsons’s |r|> 0.5, P < 0.05). Hub Gene Identification was based on intramodular connectivity (kME > 0.8).

### 2.5 Identification of AAA - associated disease targets

Intersection analysis between differentially expressed genes (DEGs)/WGCNA hub genes and the predicted targets of Aristolochic Acid A identified core AAA-associated RCC targets, which were visualized by using Venn diagrams.

### 2.6 Functional enrichment analysis

The clusterProfiler package was utilized to perform Gene Ontology (GO) analyses (biological process, cellular component, molecular function) and Kyoto Encyclopedia of Genes and Genomes (KEGG) pathway analyses (P < 0.05), elucidating the mechanistic roles of AAA in the pathogenesis of RCC.

### 2.7 Machine learning-based core gene screening

A comprehensive machine learning prediction framework that integrating multiple algorithms was applied to screen for core targets. Two datasets (GSE40435 and GSE53757) were used as the training cohort, and two independent datasets (GSE15641 and GSE68417) served as external validation cohorts. Utilizing expression profile data from these discovery and validation cohort datasets, we employed ten classical machine learning algorithms, including Lasso, SVM, RF, glmBoost, Stepglm, Ridge, Enet, GBM, LDA, XGBoost, and NaiveBayes, to develop 113 predictive models. To mitigate overfitting, the following strategies were implemented. Cross-validation for hyperparameters were optimized using ten-fold stratified cross-validation on the training cohort. Final parameters were selected based on minimal cross-validation error. Regularization. Algorithm-specific regularization was applied. For linear models, Lasso, Ridge, and Elastic Net were implemented using glmnet with a binomial family. For tree-based methods, model complexity was constrained by limiting tree depth and minimum samples per node. Early stopping was implemented by selecting the iteration number with the lowest cross-validation error, and optimal nrounds determined from cross-validation. Two datasets (GSE40435 and GSE53757) were used as the training cohort, while the independent test sets (GSE15641 and GSE68417) were served as external validation cohorts and were used only for final performance evaluation to assess generalizability. Model performance was rigorously evaluated based on the area under the ROC curve (AUC), accuracy, and F1-score. The optimal single-model predictions were subsequently integrated using a stacking ensemble learning strategy. High-confidence models (AUC > 0.9) were selected, and their feature genes were ranked by frequency to identify candidate core genes. Finally, gene expression patterns were visualized using the pheatmap package.

### 2.8 Machine learning model interpretation

Given the inherent “black box” nature of machine learning models, we implemented the SHAP (SHapley Additive exPlanations) algorithm to quantify the contribution of each feature to the predictive outcomes. This approach assigns a SHAP value to each feature, enabling interpretable assessment of its influence on the model’s predictions.

### 2.9 Deep learning-based core gene screening

A deep learning model framework was developed using the key targets identified by the AAA-predicted proteins in RCC as data sources. The analysis was performed on a total of 487 samples with 71 gene features. To ensure unbiased evaluation, the dataset was split into training (80%) and test (20%) sets using scikit-learn’s train_test_split function in Python (version 3.11) with stratification by class labels. The training set (n = 389) was then further divided into a training subset (n = 292) and a validation set (n = 97). The independent test set (n = 98) was held out for final evaluation. The network was constructed with fully connected hidden layers using the ReLU activation function. To mitigate overfitting, multiple regularisation strategies were applied at L2 weight regularisation, batch normalisation after each hidden layer, and dropout with a rate of 0.3. The model was trained using the cross-entropy loss function and the Adam optimiser for a maximum of 75 epochs, with early stopping based on validation loss (no improvement for 10 consecutive epochs). For gene correlation analysis, the Pearson correlation coefficient was applied, with thresholds set at p < 0.05 and |r| ≥ 0.3, and the NetworkX library was utilized for network visualization and metric analysis. Model interpretability was achieved through SHAP on the modified network, while comparing Transformer, CNN, Hybrid-Transformer-CNN, and BiLSTM models, all of which were trained using CrossEntropyLoss and an early stopping strategy.

### 2.10 Molecular docking analysis and molecular dynamics simulation

Molecular docking procedures were performed using CB-DOCK2, and the results were analyzed and visualized within the CB-DOCK2 interface (https://cadd.labshare.cn/cb-dock2/php/index.php). Ligand structures in SDF format and 3D protein models of core targets were retrieved from PubChem (CID:2236) and UniProt, respectively. Protein structures were preprocessed by removing water molecules and adding hydrogen atoms, while ligands underwent geometry optimization using the MMFF force field (Liu et al., 2022).

Molecular dynamics simulation for 100 ns was performed using GROMACS. The initial structure of the AAA-PYGL complex was obtained from molecular docking. The protein was parameterized with the AMBER ff14SB force field, and the ligand with GAFF2 using RESP charges. The system was solvated in a TIP3P water box, neutralized with Na^+^/Cl^−^ ions, and subjected to energy minimization followed by NVT/NPT equilibration. A 100 ns production run was conducted, with trajectories saved every 10 ps for analysis of RMSD (Root Mean Square Deviation), RMSF (Root Mean Square Fluctuation), Rg (Radius of Gyration), SASA (Solvent Accessible Surface Area), Hydrogen bonds, and MM/GBSA (Molecular Mechanics/Generalized Born Surface Area) binding free energy.

### 2.11 Mammalian cell culture

The 786-O and HK-2 cell lines, which were obtained from Dr. Chen and originally purchased from the American Type Culture Collection (ATCC), were cultured in Dulbecco’s modified Eagle’s medium (DMEM) supplemented with 10% fetal bovine serum (FBS) and 1% penicillin/streptomycin at 37^◦^ in a humidified 5% CO_2_ atmosphere.

### 2.12 Drug treatment

Aristolochic Acid A (HY-N0510) and glycogen phosphorylase (PYGL) inhibitor CP-91149 (HY-13525) were obtained from MedChemExpress (MCE), China. HK-2 cells were seeded into six-well plates. After 24 h, the cells were treated with varying concentrations of AAA (Upadhyay and Batuman, 2022; Liu Z. et al., 2025). CP-91149 or an equal volume of dimethyl sulfoxide (DMSO) as a Vehicle control (Li et al., 2024).

### 2.13 Western blot analysis

Standard western blotting procedures were used (Qi et al., 2021). Cells were lysed in radioimmunoprecipitation assay (RIPA) buffer containing proteinase inhibitor and phosphatase inhibitor cocktail. Protein concentration was determined by a Pierce BCA Protein Assay Kit (Thermo Fisher Scientific, United States of America). Equal amounts of protein were separated by sodium dodecyl sulfate-polyacrylamide gel electrophoresis (SDS-PAGE), transferred to polyvinylidene difluoride (PVDF) membrane, and detected by chemiluminescence. GAPDH served as the internal loading control. Antibodies used included anti-GAPDH (Abl1020, Abbkine, China), anti-PYGL (15851-1-AP, Proteintech, China), and anti-E-cadherin (AF648, R&D, United States of America). Densitometric analysis of the protein bands was performed using ImageJ software (National Institutes of Health, Bethesda, MD, United States of America). The intensity of each protein band (either PYGL or E-cadherin) was normalized to that of the loading control (GAPDH), and the results were expressed as the fold change relative to the respective control group (set as 1.0).

### 2.14 PYGL gene knockdown via siRNA transfection

To knock down PYGL expression, two independent PYGL-specific small interfering RNAs (siRNA) were used (GenePharma, Shanghai, China), with a scrambled siRNA serving as the negative control (Li et al., 2024). PYGL siRNAs were transfected into HK-2 cells using the siRNA-Mate SUS kit and its associated delivery system reagents (G04037, GenePharma, Shanghai, China) according to the manufacturer’s protocol. Negative control (NC siRNA) and two PYGL siRNAs were synthesized from GenePharma company.

Human PYGL siRNA-1774: sense:5’-CCUGGAGACGGAGUACAAATT-3’ and antisense: 5’-UUU​GUA​CUC​CGU​CUC​CAG​GTT -3’.

Human PYGL siRNA-548: Sense:5’-GCUGCCUGCUUCUUGGAUUTT -3’ and antisense: 5’-AAU​CCA​AGA​AGC​AGG​CAG​CTT-3’; and the scramble sequences 5’-UUC​UCC​GAA​CGU​GUC​ACG​UTT -3’ and 5’-ACG​UGA​CAC​GUU​CGG​AGA​ATT -3’ serving as negative control.

### 2.15 Statistical analysis

Renal cell carcinoma (RCC) transcriptomic data were processed using R software (version R.4.3.3). All biological data were derived from at least three independent experiments and data are presented as the means ± standard errors of the mean (SEMs). GraphPad Prism 10 Statistics software (Version 10.2.0) was unitized for conducting statistical analysis. Comparisons between two groups were analyzed using Student’s t-test for unpaired comparisons. For multiple group comparisons, one-way ANOVA was used, followed by *post hoc* analysis. Significance levels were indicated as: *p < 0.05, **p < 0.01, ***p < 0.001, and ****p < 0.0001. The threshold for statistical significance was set at p < 0.05.

**FIGURE 1:**
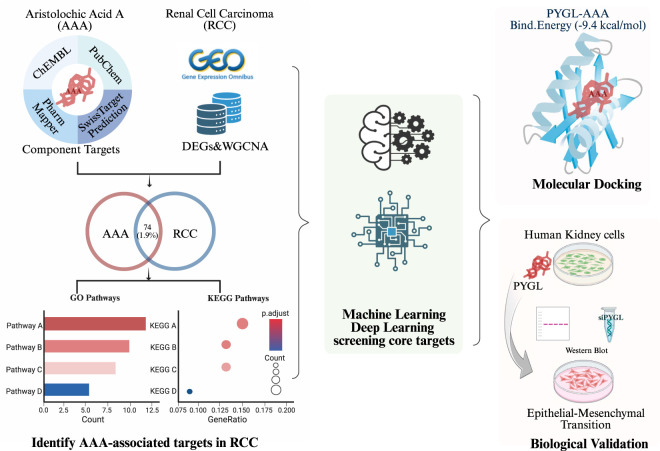
Schematic outline of data analysis process in this study.

The complete analytical workflow is illustrated in Figure 1.

## 3 Results

### 3.1 Identification of aristolochic acid A (AAA) target proteins

**FIGURE 2:**
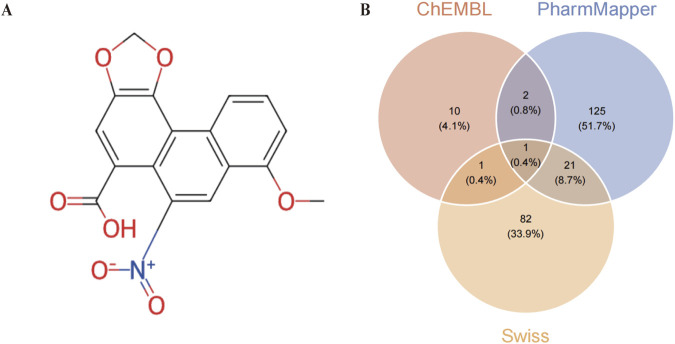
Identification of Aristolochic Acid A (AAA) Target Proteins **(A)** Chemical structure of AAA. **(B)** Target prediction using CHEMBI, PharmMapper, andSwissTargetPrediction.

The molecular structure of Aristolochic Acid A (AAA) was retrieved from the PubChem database (Figure 2A). Potential biological targets of AAA were systematically predicted using three complementary databases: ChEMBL (curated bioactive molecules), PharmMapper (reverse pharmacophore mapping), and SwissTargetPrediction (ligand-based target prediction). After data integration and the removal of redundant entries, a total of 242 unique potential targets were identified (Figure 2B).

### 3.2 Identification of RCC-related target genes

**FIGURE 3:**
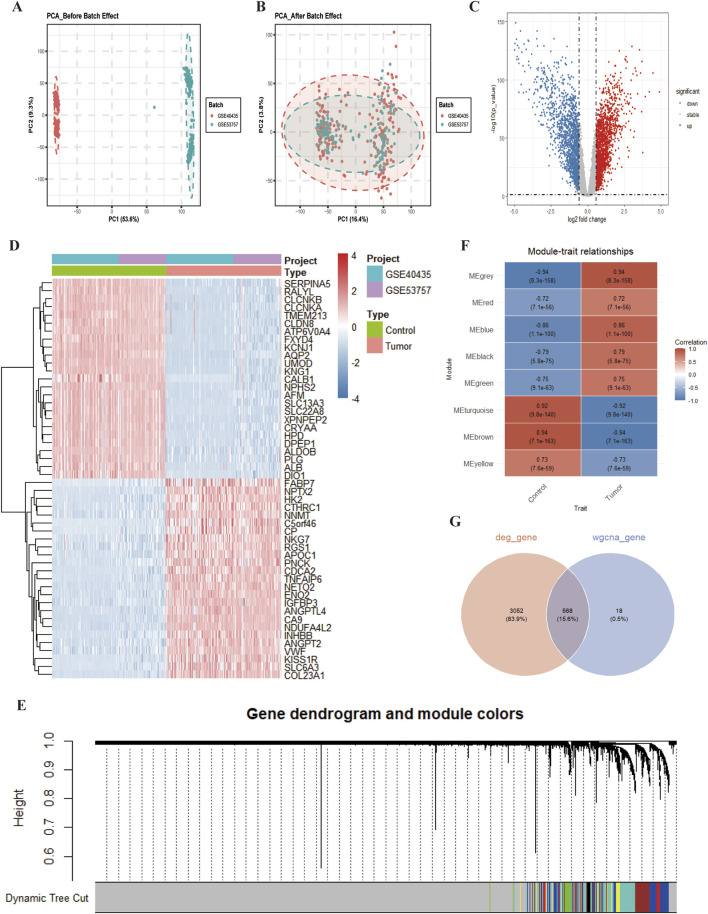
Identification of RCC-Related Target Genes **(A)** PCA scatter plot shows distinct separation between GSE40435 and GSE53757 datasets before batch correction, indicating batch effects. **(B)** PCA scatter plot after batch correction shows the integration of GSE40435 and GSE53757 datasets, indicating reduced batch effects. **(C)** Volcano plot shows DEGs based on log2FoldChange and significance. Red dots are upregulated, blue dots are downregulated, and grey dots are non-significant. **(D)** Heatmap shows DEG expression patterns across samples. Coral red indicates upregulation; light blue indicates downregulation. **(E)** Gene dendrogram from WGCNA shows hierarchical clustering based on co-expression. Module colors in the lower panel represent different gene modules. **(F)** Module-trait relationships heatmap shows correlations between WGCNA-identified modules and sample traits (Control vs. Tumor). Values in boxes indicate correlation coefficients and p-values. **(G)** Venn diagram shows degs (coral orange) and wgcna modules (light blue), with Apricot indicating common genes from both methods.

To minimize batch effects, we merged the GSE40435 and GSE53757 datasets and performed comprehensive normalization of the gene expression matrices. Principal component analysis (PCA) demonstrated improved data distribution following normalization, with the normalized dataset exhibiting more distinct clustering patterns (Figures 3A,B). Differential expression analysis identified 3,620 genes with significant alterations in renal cell carcinoma (RCC). These expression changes were visualized through volcano plots and heatmaps (Figures 3C,D). For weighted gene co-expression network analysis (WGCNA), we determined the optimal soft-thresholding power (β) to ensure scale-free network topology. Systematic evaluation of power values ranging from 1 to 20 revealed that β = 19 was the minimum value achieving the scale-free topology criterion (R^2^ ≥ 0.8). Using this parameter, we constructed a topological overlap matrix (TOM) and performed hierarchical clustering to identify co-expression modules. This analysis yielded eight distinct gene modules, each color-coded for visualization (Figure 3E). Module-trait relationship analysis identified significant associations (P < 0.05) between specific modules and RCC (Figure 3F). By integrating differentially expressed genes from conventional analysis with module genes from WGCNA (after removing duplicates), we identified a final set of 3,638 RCC-associated genes (Figure 3G).

### 3.3 Identification of AAA-associated disease targets in RCC

**FIGURE 4:**
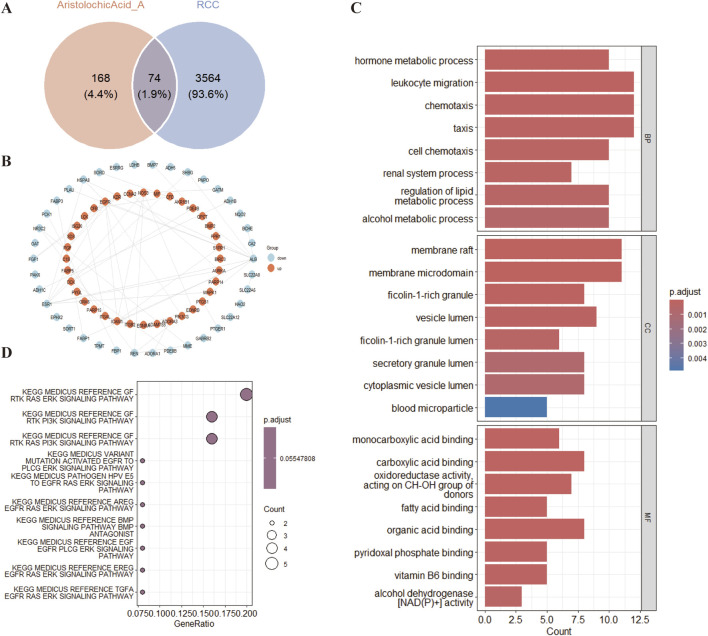
Identification of AAA-Associated Disease Targets in RCC **(A)** Venn diagram compares genes linked to AAA exposure (red) and RCC (blue), with 74 overlapping genes (1.9%). **(B)** PPI network visualizes interactions among overlapping genes. Red nodes, upregulated; light blue nodes, downregulated; edges, predicted interactions. **(C)** GO enrichment annotates overlapping genes in BP, CC, and MF. X-axis, gene count; color gradient, adjusted p-value (darker red, higher significance). **(D)** KEGG analysis shows enriched pathways for overlapping genes. X-axis, gene ratio, dot size, gene count; color gradient, adjusted p-value (red, higher significance).

The intersection analysis between AAA target proteins and RCC-related genes identified 74 potential key targets involved in AAA-induced RCC (Figures 4A,B). Functional characterization through GO and KEGG enrichment analyses (Figures 4C,D) revealed comprehensive molecular insights. GO analysis demonstrated significant enrichment in leukocyte migration, chemotaxix (biological processes), membrane raft and membrane microdomain (cellular components), as well as carboxylic acid binding, organic acid binding and oxidoreductase activity, acting on CH-OH group of donors (molecular functions). KEGG pathway analysis highlighted GFRTK RAS/ERK signaling pathway and RAS/PI3K signaling pathway. These findings collectively indicate significant activation of cell proliferation, and survival, angiogenesis in AAA-related renal cell carcinoma.

### 3.4 Identification of core genes in AAA-induced RCC by machine learning

**FIGURE 5:**
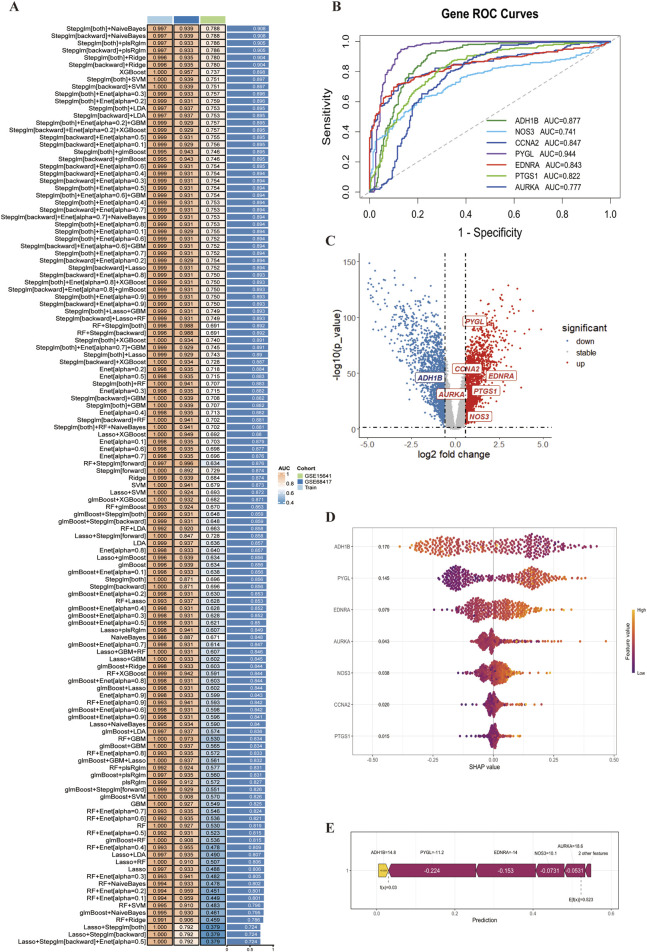
Identification of Core Genes in AAA-Induced RCC using Machine learning **(A)** heatmap of model performance comparison shows AUC values for various models across cohorts. Left column, models; right column, AUC. Colors indicate cohort sources. **(B)** ROC curves for seven cores genes (ADH1B, NOS3, CCNA2, PYGL, EDNRA, PTGS1, AURKA). X-axis, false positive rate; Y-axis, sensitivity. AUC indicates predictive performance. **(C)** Volcano plot shows DEGs. X-axis, log2 Fold Change; Y-axis, -log10 (p value). Red , upregulated; blue, downregulated, with key genes labeled. **(D)** Violin plot shows gene expression distributions across conditions. Width, data density; colors, expression levels. **(E)** SHAP summary plot of the GSM994036 sample shows gene contributions to predictions. Negative SHAP, lowering effect; positive, increasing effect.

Through a comprehensive machine learning analysis of 74 candidate targets, we developed 113 predictive models to identify core genes associated with AAA-related renal cell carcinoma (RCC). Among these models, the Stepglm [glm] + NaiveBayes ensemble model demonstrated the highest performance, exhibiting superior accuracy in both the training and validation phases (Figure 5A). This analysis led to the identification of seven pivotal genes: ADH1B, NOS3, CCNA2, PYGL, EDNRA, PTGS1 and AURKA. Receiver operation characteristic (ROC) confirmed that these core genes have good diagnostic potential, with the highest AUC greater than 0.9 for PYGL (Figure 5B). Additionally, the differential expression patterns in RCC tissues were further visualized using a volcano plot (Figure 5C). SHAP (Shapley Additive exPlanations) interpretability analysis revealed differences in the functional contributions of each gene in model predictions: ADH1B (SHAP value = 0.17) and PYGL (0.145) were the most influential predictors (Figure 5D). Force-directed analysis of the GSM994036 sample (Figure 5E) further demonstrated that ADH1B (14.8, Δ = +0.0254) served as primary positive regulator, while PYGL (−11.2, Δ = −0.224) and EDNRA (−14, Δ = −0.153) served as primary negative regulators, driving prediction values (f(x) = 0.03) below the benchmark expectation (E [f(x)] = 0.523).

### 3.5 Identification of feature genes in AAA-induced RCC by deep learning

**FIGURE 6:**
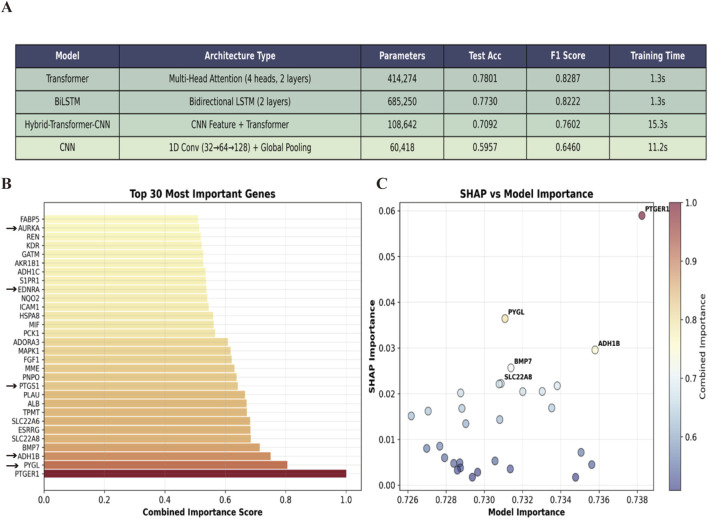
Identification of core genes in AAA-Induced RCC using deep learning **(A)** Model performance comparison table displays the Test accuracy, F1 Score, and training time for various models across cohorts. **(B)** Bar plot illustrates the top 30 most important genes identified by the deep learning algorithms. The black arrows highlight the five feature genes identified by the Machine learning algorithm. **(C)** Scatter plot depicts the relationship between SHAP importance and model importance for the identified genes, highlighting the top five represented genes.

Among four deep classification network models evaluated, the Transformer achieved the highest test accuracy at 78.01%, followed by BiLSTM with 77.30%, and CNN, which attained only 59.57%. The Transformer not only demonstrated the best overall performance with a test accuracy of 0.7801 and an F1 score of 0.8287, but it also had the fastest training time of 1.3 s (Figure 6A). Furthermore, except for CCNA2 and NOS3, five genes identified through machine learning appeared among the top 30 most important genes in the deep classification network analysis (Figure 6B). Model interpretability analysis utilizing SHAP indicated that genes such as PTGER1, PYGL, ADH1B, BMP7, and SLC22A8 have a substantial influence on the model’s predictions (Figure 6C).

### 3.6 Molecular docking validation of AAA-core genes interactions

**FIGURE 7:**
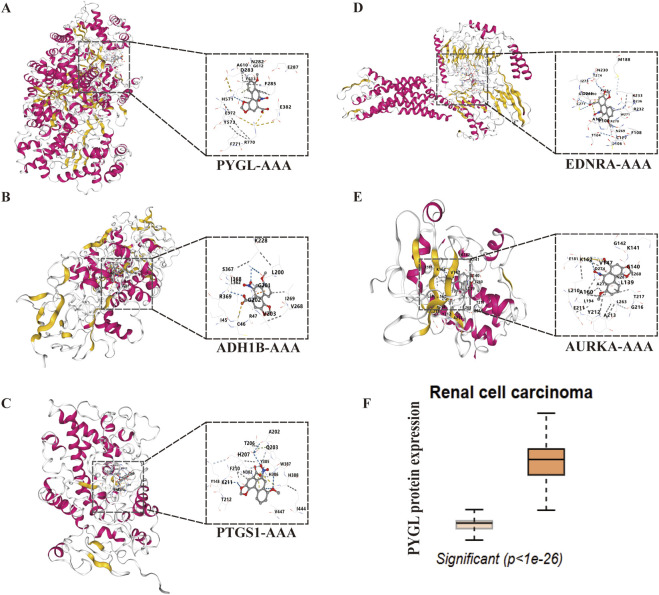
Molecular docking validation of AAA-core gene interactions **(A)** Docking results of PYGL with AAA. **(B)** Docking results of ADH1B with AAA. **(C)** Docking results of PTGS1 with AAA. **(D)** Docking results of EDNRA with AAA. **(E)** Docking results of AURKA with AAA. **(F)** PYGL protein expression levels in Renal cell carcinoma (Human Protein Atlas).

To validate the potential binding interactions between AAA and the common core genes identified through machine learning and deep learning, we conducted comprehensive molecular docking analyses for the five core genes: PYGL, ADH1B, PTGS1, EDNRA, and AURKA, as shown in Figures 7A–E. Visualization of the binding conformations revealed stable docking poses for all AAA-protein complexes. Moreover, to validate this finding, the protein expression of the identified core gene with the lowest binding energy was explored in the Human Protein Atlas. The results of PYGL protein expression levels in renal cell carcinoma are indicated by the Human Protein Atlas, as shown in Figure 7F. Furthermore, the binding energy results indicated strong binding affinities between AAA and all five target proteins, with binding energies consistently below -5 kcal/mol, and the lowest value at −9.5 kcal/mol for PYGL (Table 1). This suggests the stable and spontaneous molecular interactions between AAA and the identified core gene regulators, highlighting their biomarker and therapeutic potential.

**TABLE 1:** Binding energies of ligands and core gene regulators.

Ligand	Core gene regulator	Bind. energy [kcal/mol]
Aristolochic acid A	PYGL	−9.4
Aristolochic acid A	EDNRA	−8.6
Aristolochic acid A	AURKA	−8.5
Aristolochic acid A	ADH1B	−7.9
Aristolochic acid A	PTGS1	−7.9

### 3.7 Molecular dynamics simulation of AAA-PYGL interaction stability

**FIGURE 8:**
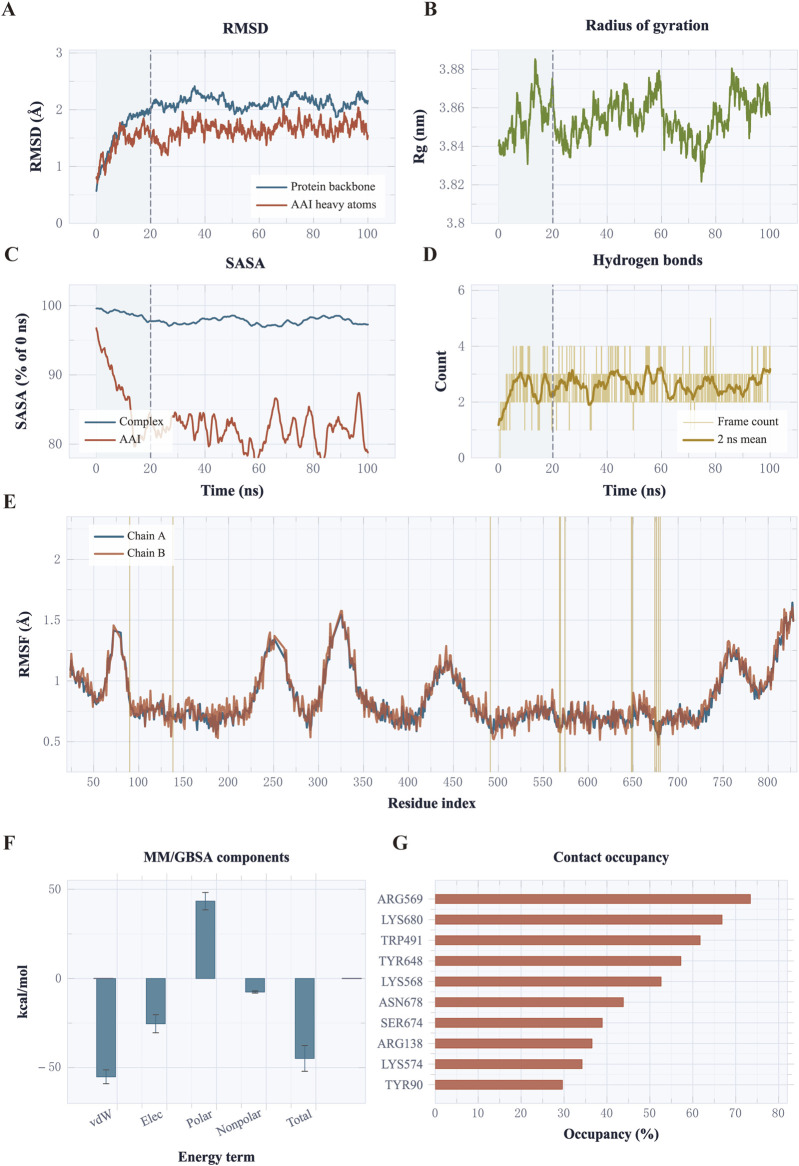
Molecular Dynamics Simulation of AAA-PYGL Binging Stability. **(A)** Time evolution of RMSD trajectory of PYGL protein and the AAA-PYGL complex. **(B)** Radius of gyration (Rg) of the PYGL dimer during the 100 ns MD simulation. **(C)** SASA during the 100 ns MD simulation for AAA and its PYGL complex. **(D)** Hydrogen bond count between AAA and PYGL during the 100 ns simulation. **(E)** Per-residue RMSF of PYGL. Gold vertical bars indicate pocket-proximal residues surrounding the PLP binding site. Chain A (teal) and Chain B (salmon) exhibited comparable fluctuation profiles, with lower RMSF values in the ligand-binding pocket region. **(F)** MM/GBSA energy decomposition for the AAA-PYGL complex. Energy components include van der Waals (vdW), electrostatic (Elec), polar solvation (Polar), nonpolar solvation (Nonpolar), and total binding free energy (Total). Error bars represent standard deviations from 20 to 100 ns frame analysis. **(G)** Contact occupancy of key pocket residues with AAA during the equilibrium phase. Residues are ranked by occupancy percentage, with ARG569, LYS680, TRP491, and TYR648 showing the highest persistent contacts.

The AAA-PYGL binding complex remained stable throughout the 100 ns simulation with the initial molecular docking score at −9.4 kcal/mol, as shown in Figures 8A–G. The protein backbone RMSD reached a plateau after 20 ns, averaging 2.13 ± 0.10 Å during the equilibrium phase, while the ligand heavy-atom RMSD averaged 1.65 ± 0.14 Å, indicating that AAA maintained its binding pose (Figure 8A). The radius of gyration remained stable at 3.85 ± 0.01 nm, consistent with the preserved compactness of the functional PYGL (Figure 8B). The ligand SASA decreased markedly during the initial 20 ns and stabilized at a lower level, indicating progressive burial of AAA into the PYGL binding pocket (Figure 8C). An average of 2.64 ± 0.65 hydrogen bonds were maintained between AAA and PYGL during the equilibrium phase (Figure 8D). RMSF analysis showed that the pocket-proximal residues (gold bars) maintained low flexibility, indicating a relatively rigid ligand-binding region consistent with stable AAA-PYGL complex formation (Figure 8E). MM/GBSA calculations revealed a favorable total binding free energy (ΔG_bind) of −44.8 ± 7.2 kcal/mol, driven by van der Waals and electrostatic interactions, partially offset by polar solvation (Figure 8F). Key residues including ARG569, LYS680, TRP491, and TYR648 showed high contact occupancy with AAA, forming persistent polar and hydrophobic interactions (Figure 8G). These results demonstrate that AAA forms a stable and energetically favorable complex with PYGL, consistent with the docking-predicted strong affinity.

### 3.8 *In vitro* validation of PYGL as a target of AAA-induced RCC in human kidney cells

**FIGURE 9:**
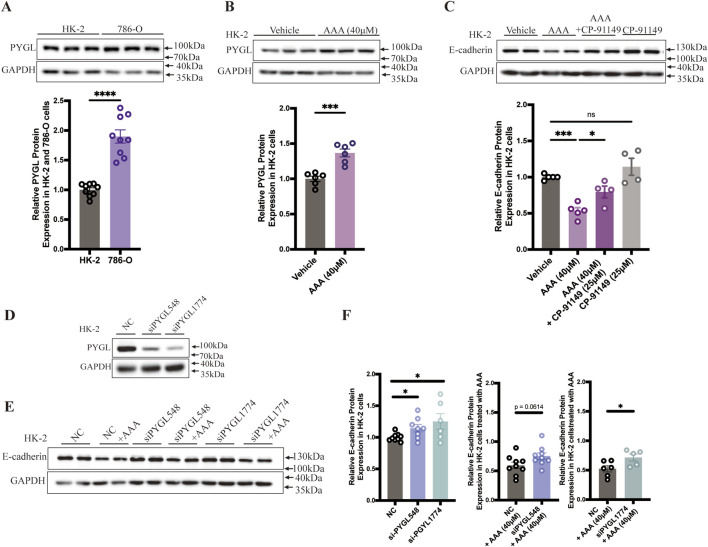
Validation of PYGL as a potential toxic target in AAA-induced epithelial injury in human kidney cells **(A)** Representative immunoblots and quantitative analysis of PYGL protein levels in HK-2 (renal proximal tubular epithelial) and 786-O (clear cell adenocarcinoma) cells. Protein levels were normalized to GAPDH. **(B)** Representative immunoblots and quantitative analysis of PYGL protein levels in HK-2 cells treated with AAA (40 μM) or vehicle (0.1% DMSO) for 24 h. **(C)** Representative immunoblots and quantitative analysis of E-cadherin protein levels in HK-2 cells treated with vehicle (0.1% DMSO), AAA (40 μM), AAA combined with the glycogen phosphorylase inhibitor CP-91149 (25 μM), or CP-91149 alone for 24 h. **(D)** Representative immunoblots showing PYGL protein levels in HK-2 cells transfected with scrambled siRNA (negative control, NC), siPYGL-548, or siPYGL-1774 for 24 h. PYGL levels were normalized to GAPDH and expressed as fold change relative to NC. **(E,F)** Representative immunoblots and quantitative analysis of E-cadherin protein levels in HK-2 transfected with scrambled siRNA (negative control, NC) or siPYGL-548, siPYGL-1774 for 24 h. E-cadherin levels were normalized to GAPDH and expressed as fold change relative to NC. E-cadherin protein levels in HK-2 cells treated with AAA (40 μM) combined with siPYGL-548 or scrambled siRNA (NC) for 24 h. E-cadherin levels were normalized to GAPDH and expressed as fold change relative to AAA + NC. Protein levels were normalized to GAPDH and expressed as fold change relative to individual control. Data are presented as mean ± SEM. N = 3, * P < 0.05, ** P < 0.01, *** P < 0.001, ns: no significant.

By integrating machine learning, deep learning, molecular docking, and dynamic simulation analyses, the top-ranked candidate, PYGL, was selected for further biological function validation in normal human kidney cells (HK-2) and renal cell carcinoma cells (786-O), as shown in Figure 9. PYGL protein expression was assessed in the normal human kidney cell line HK-2 and the renal cell carcinoma cell line 786-O. As shown in Figure 9A, PYGL protein levels were significantly higher in 786-O cells than in HK-2 cells (fold change = 1.898, p < 0.0001), as determined by densitometric analysis.

To test our hypothesis that AAA interacts with PYGL, the top-ranked molecule potentially implicated in the AAA-induced RCC, PYGL protein expression was assayed in HK-2 cells treated with AAA or vehicle alone. As shown in Figure 9B, PYGL protein levels were significantly increased after 24 h of treatment with 40 µM AAA compared with that of the vehicle control. Furthermore, to investigate whether increased PYGL exerts a negative effect in AAA-treated HK-2 cells, we evaluated the protein level of E-cadherin, an epithelial-mesenchymal-transition marker indicating a shift toward a mesenchymal phenotype. E-cadherin levels were decreased in AAA-treated HK-2 cells compared with the Vehicle control group. Moreover, this reduction in E-cadherin protein levels was markedly reversed by CP-91149 (25 µM), a PYGL inhibitor, whereas CP-91149 (25 µM) alone had no significant effect on E-cadherin protein expression (Figure 9C).

In addition, two siRNA constructs targeting PYGL were transfected into HK-2 cells, with negative control (NC) siRNA serving as the control. Western blot assays showed that both siRNAs markedly decreased PYGL protein expression (Figure 9D). Furthermore, PYGL siRNA knockdown exhibited a small but significant increase in E-cadherin expression under basal conditions (Figures 9E,F). When exposed to AAA (40 µM) for 24 h, E-cadherin protein levels were substantially restored in PYGL siRNA-transfected cells compared with the scramble siRNA negative control. While siPYGL-548 group yielded a p-value of 0.0614, which did not reach the predefined threshold, the other siRNA (siPYGL-1774) achieved statistical significance (p < 0.05) (Figures 9E,F). Notably, both siRNAs targeting distinct PYGL sequences showed a clear and consistent trend toward E-cadherin rescue, arguing against off-target artifacts and supporting the specificity of the observed phenotype. Since decreased E-cadherin reflects acquisition of a more mesenchymal phenotype, this partial rescue upon PYGL knockdown indicates that PYGL contributes, at least in part, to this phenotypic transition.

In summary, our *in vitro* studies demonstrate that either PYGL knockdown or PYGL inhibitor treatment restores E-cadherin expression and reversed the EMT-associated epithelial phenotype loss, suggesting that PYGL acts as a key regulatory of AAA-induced toxicity.

## 4 Discussion

In this study, we employed an integrated framework combining machine learning, deep learning, molecular docking, and *in vitro* validation to uncover molecular targets linking Aristolochic acid A (AAA) to renal cell carcinoma (RCC), identifying PYGL as a novel functional mediator. This approach demonstrates a feasible and efficient strategy for elucidating carcinogen-target interactions in toxicological studies, particularly for diseases with complex etiology and limited clinical specimens.

AAA is a recognized carcinogen, with its nephrotoxic and carcinogenic properties linked to aristolochic acid-associated nephropathy (Ji et al., 2021) and cancers of the kidney and liver (Zhang et al., 2024; Zhang et al., 2025 B.). In 2022, the International Agency for Research on Cancer (IARC) formally classified aristolochic acids as carcinogenic to humans based on comprehensive evidence of their mutagenicity and carcinogenicity (Das et al., 2022), highlighting the ongoing global health threat posed by continued exposure through inadequately regulated herbal products.

Multiple mechanisms contribute to AAA-induced nephrotoxicity, including direct cytotoxicity, metabolic activation, DNA damage and mutagenicity, oxidative stress, and inflammatory responses with immune cell infiltration (Yang et al., 2018; Anger et al., 2020; Chen et al., 2022; Zhou et al., 2023; Zhang et al., 2024). Despite these advances, the precise molecular pathways driving AAA-associated carcinogenesis, particularly in RCC, remain incompletely understood (Anger et al., 2020).

Recent advances in bioinformatics and artificial intelligence, particularly machine learning and deep learning, have facilitated the rapid discovery of potential genetic and molecular targets (Liu et al., 2020; Chen et al., 2023; Ballard et al., 2024; Fan, 2025; Wang et al., 2025; Gao et al., 2025a). Leveraging these emerging predictive tools with established network pharmacology approached, we integrated multi-omics data with machine learning, deep learning, network toxicology, and molecular docking to prioritize candidate targets. Our approach screened 74 candidate genes and identified seven core genes (ADH1B, PYGL, NOS3, CCNA2, EDNRA, PTGS1, and AURKA) as key players. Differential expression analysis revealed that ADH1B is consistently downregulated in RCC, whereas PYGL, NOS3, CCNA2, EDNRA, PTGS1, and AURKA are significantly upregulated. Machine learning models further validated the diagnostic utility of this gene set, with SHAP interpretability analysis highlighting ADH1B (SHAP value = 0.170) and PYGL (SHAP value = 0.145) as critical predictors.

Subsequently, deep learning-based feature selection independently prioritized five of these seven genes including ADH1B, PYGL, PTGS1, EDNRA, and AURKA. These deep learning models achieved high accuracy in gene expression prediction (up to 98.98%), underscoring the effectiveness of feature dimensionality reduction and data augmentation techniques. Correlation analysis revealed pivotal roles for hub genes like PTGER and PYGL in the network, with SHAP-based interpretability analysis highlighting the strong influence of PTGER1, PYGL, and ADH1B. Both machine learning and deep learning results consistently identified PYGL and ADH1B as having the greatest predictive power.

Moreover, these five convergent genes were advanced to molecular docking analyses to evaluate the binding affinities between AAA and their encoded protein products. All five proteins exhibited relative strong binding activity (binding energies below −7.0 kcal/mol), with PYGL showing the strongest affinity at −9.4 kcal/mol.

The biological relevance of these five identified genes in RCC is supported by existing literature. ADH1B encodes alcohol dehydrogenase 1B, a key enzyme in alcohol metabolism that inhibits tumorigenesis by reducing alcohol intake and promoting retinoic acid production, thereby regulating cell growth and differentiation. A study discovered that in patients with RCC, serum total ADH activity and class I isoenzyme activity were significantly elevated across all tumor stages compared to healthy controls. Given that ADH1B is a core member of the class I ADH family, this study provides direct support for its diagnostic and prognostic potential in RCC (Orywal et al., 2018). Additionally, genome-wide association studies (GWAS) have linked polymorphisms in the 4q23 chromosomal region (e.g., ADH1B rs1229984 variant) to increased RCC risk (Dračínská et al., 2016; Govind et al., 2020).

Furthermore, PYGL, as the top-ranked identified gene, encodes glycogen phosphorylase, which is a rate-limiting enzyme in glycogenolysis. Earlier data from the Human Protein Atlas found that PYGL protein expression was elevated in RCC patients (Zhang X. et al., 2025), but it was not prognostic in clear cell RCC. However, Li et al. (2024) recently discovered that PYGL is significantly upregulated at both mRNA and protein levels in ccRCC, and integrated genomic and proteomic analyses identified PYGL as a novel therapeutic target (Li et al., 2024). Mechanistically, HIF-1α-mediated hypoxia drives PYGL expression, promoting tumor proliferation, metastasis, and disease progression through glycogen metabolism and necroptosis. Notably, targeting PYGL shows direct antitumor effects and overcomes sunitinib resistance. Moreover, PYGL overexpression correlates with advanced tumor staging, higher grading, reduced overall survival (OS), and progression-free survival (PFS) (Favaro et al., 2012; Li et al., 2024). Consistent with these findings in RCC, another study discovered that PYGL-mediated glucose metabolism reprogramming promotes EMT phenotype in the progress of pancreatic cancer metastasis (Ji et al., 2023). This evidence strengthens that the role of PYGL as a potential prognostic biomarker.

In addition, PTGS1 encodes cyclooxygenase-1 (COX-1), the rate-limiting enzyme in prostaglandin (PG) synthesis. Through the production of PGE2, COX-1 may promote RCC by enhancing tumor cell proliferation, survival, angiogenesis, and immune evasion. Analysis of 64 primary RCC cases revealed that high COX-1 levels correlate with tumor grade, size, stage, invasion, and co-expression with vascular endothelial growth factor (VEGF), aiding in cancer staging predictions (Osman and Youssef, 2015). Although direct mechanistic studies of PTGS1 in RCC remain limited, a recent transcriptomic analysis of the arachidonic acid pathway reported that PTGS1 (COX-1) is significantly upregulated in RCC, particularly in patients without obesity or smoking comorbidities. This finding is consistent with the established association between COX-1 expression and clinicopathological features of RCC (Aradhyula et al., 2024).

EDNRA, another identified overlapping gene, encodes endothelin receptor type A, which is highly expressed in RCC and associates with poor prognosis. It activates pathways like JAK-STAT and TGF-β, promoting immune evasion through interactions with immunosuppressive factors such as ADORA2A, CSF1R, and TGFB1 (Wang et al., 2024). Beyond these pathways, the EDN1-EDNRA-OLR1 signaling axis serves as a central mediator of immunosuppressive crosstalk. EDNRA is positioned at the hub of a hypoxia-ETS1-nucleolar stress-macrophage axis, revealing its critical role in shaping the immunosuppressive tumor microenvironment and driving ccRCC progression (Xiao et al., 2025).

Lastly, AURKA encodes a serine/threonine kinase that facilitates spindle formation and chromosome segregation. Its dysregulation exacerbates tumor malignancy by compromising chromatin stability (Li et al., 2021). Wen et al. demonstrated that AURKA level positively correlated with RCC pathological staging and inversely correlated with patient survival (Wen et al., 2024). Beyond its role in cell cycle regulation, it has been demonstrated that AURKA promotes tumor cell proliferation, migration, and invasion while also significantly modulating anti-tumor immunity. Consequently, AURKA has been identified as a potential therapeutic target with clear translational value (Ma et al., 2026).

Collectively, direct evidence specifically linking these identified genes to AAA-induced RCC is currently lacking. However, their established roles in general RCC, together with their identification through our screening method, provide a strong rationale for hypothesizing their involvement in AAA-related renal carcinogenesis.

To further validate the core target prior to in vitro studies, molecular dynamics simulation was performed to assess the binding stability of AAA with PYGL. The results revealed that AAA forms a stable and energetically favorable complex with PYGL over the simulation trajectory, supported by persistent hydrogen bonds, aromatic stacking, and electrostatic interactions within the binding pocket. These findings are consistent with the docking- predicted strong affinity and reinforce the reliability of the binding model. On this basis, we proceeded to *in vitro* studies validate the functional role of PYGL in AAA-induced renal cell injury using HK- 2 cells and the RCC cell line 786-O. In our study, PYGL expression was significantly elevated in 786-O cells (a clear cell RCC cell line) compared with normal human kidney HK-2 cells. Moreover, AAA treatment at 40 μM induced a marked increase in PYGL protein expression in HK-2 cells. Our preliminary data showed that 100 μM AAA induced significant cytotoxicity in HK-2 cells, whereas at 40 μM AAA we observed a mild to moderate increase in cytotoxicity. Consistent with Upadhyay and Batuman, 2022, 40 μM AAA does cause some degree of cell injury, the predominant effects are cell cycle arrest, proliferation inhibition, and cellular senescen (Gao P. et al., 2025b; Gao et al., 2026). Nevertheless, Upadhyay and Batuman, 2022 also confirmed that 40 μM AAA effectively induces EMT in HK-2 cells, as evidenced by decreased E-cadherin and increased vimentin and α-SMA expression (Upadhyay and Batuman, 2022). Therefore, the 40 µM condition used in this study modeled early, senescence and EMT-related molecular changes.

It is known that reduced E-cadherin indicates a transition of renal tubular cells from an epithelial to a mesenchymal phenotype, a process potentially associated with carcinogenesis. This aligns with previous studies showing that AAA time-dependently reduces E-cadherin levels in kidney cells, promoting a shift from a normal epithelial phenotype toward a mesenchymal state. In our study, AAA treatment markedly reduced E-cadherin expression in HK-2 cells. Notably, either pharmacological inhibition or genetic knockdown of PYGL restored E-cadherin expression, supporting a role for PYGL in epithelial-mesenchymal transition (EMT). Ji et al. (2023) reported that PYGL promotes EMT and metastasis in pancreatic cancer ductal adenocarcinoma (Ji et al., 2023), and Li et al. (2024)identified PYGL as an oncogene involved in EMT-like changes and sunitinib resistance in clear cell RCC (Li et al., 2024). Consistent with these results, our findings demonstrate that PYGL inhibition reverses AAA-induced E-cadherin downregulation. Together with the pharmacological inhibition of PYGL by its inhibitor, these findings suggest that PYGL acts as a key regulator of AAA-induced EMT in renal tubular epithelial cells. This in vitro validation confirms that AAA upregulates PYGL expression, and that PYGL knockdown partially reverses AAA- induced EMT, as evidenced by E-cadherin restoration.

Our integrated framework and in vitro data suggest that PYGL serve as a key target in AAA-induced RCC and illustrate how AI- assisted approaches can shorten the target identification cycles.

According to two recent reviews (Tran et al., 2023; Chakraborty et al., 2024), the major challenges of AI-assisted drug toxicity prediction are the lack of high-quality, diverse training data and limited interpretability of deep learning models. Consistent with these broader concerns, our study also has several limitations. Our data were derived from RCC transcriptomic datasets in the NCBI GEO database and did not integrate other omics sources. Second, the sample size, while sufficient for exploratory analysis, may limit generalizability. Third, this study focused exclusively on the toxicity mechanisms of AAA, excluding metabolic intermediates such as Aristolactam nitrenium ions (Yun et al., 2015). Finally, due to time and resource constraints, both molecular dynamics simulation and in vitro validation were limited to PYGL, the top-ranked target, leaving other core targets unexamined.

To address these limitations, future research should expand cohorts, incorporate diverse samples (e.g., high AAA-exposure populations), and conduct additional *in vitro* and *in vivo* validations. Comprehensively validation using a combination of techniques including cellular thermal shift assays (CETSA), surface plasmon resonance (SPR), mass spectrometry-based small-molecule interaction profiling, site-directed mutagenesis, and *in vivo* models, will be pursued to elucidate how AAA directly targets PYGL to promote RCC pathogenesis. Moreover, elucidating the molecular interactions between these targets and the AAA network, especially at the levels of epigenetic modifications and protein interactomes, will provide a critical theoretical foundation for developing effective therapies.

In conclusion, this study demonstrates the power of integrating multi-omics data, AI-driven computational tools, and *in vitro* validation to uncover the mechanisms underlying AAA-induced RCC, highlighting a pioneering hybrid approach that seamlessly integrates computational predictive modeling with wet-lab assays.

## 5 Conclusion

In conclusion, this study elucidates that Aristolochic acid A (AAA) may contribute to renal cell carcinoma pathogenesis by targeting specific molecules. Using an integrated approach of machine learning, deep learning, and molecular docking, we identified five core regulatory genes. Subsequently, molecular dynamics simulation and *in vitro* studies validated the top-ranked candidate, PYGL. This integrative strategy offers a faster and more efficient alternative to conventional methods, providing a feasible solution for toxicological research on herbal compounds, especially when access to clinical specimens is limited.

## Data Availability

The original public datasets used as the basis of this reanalysis are available in the Gene Expression Omnibus (GEO) database under accession numbers of GSE40435 (https://www.ncbi.nlm.nih.gov/geo/query/acc.cgi?acc=GSE40435), GSE53757 (https://www.ncbi.nlm.nih.gov/geo/query/acc.cgi?acc=GSE53757), GSE15641 (https://www.ncbi.nlm.nih.gov/geo/query/acc.cgi?acc=GSE15641), and GSE68417 (https://www.ncbi.nlm.nih.gov/geo/query/acc.cgi?acc=GSE68417). The data presented in this study are deposited in the GitHub repository at https://github.com/StumbleMQ/Aristolochic-Acid-A-Carcinogenesis-Prediction.

## References

[B1] Anger E. E. Yu F. Li J. (2020). Aristolochic acid-induced nephrotoxicity: molecular mechanisms and potential protective approaches. Int. J. Mol. Sci. 21, 1157. 10.3390/ijms21031157 32050524 PMC7043226

[B2] Aradhyula V. Breidenbach J. D. Khatib-Shahidi B. Z. Slogar J. N. Eyong S. A. Faleel D. (2024). Transcriptomic analysis of arachidonic acid pathway genes provides mechanistic insight into multi-organ inflammatory and vascular diseases. Genes 15, 954. 10.3390/genes15070954 39062733 PMC11275336

[B3] Ballard J. L. Wang Z. Li W. Shen L. Long Q. (2024). Deep learning-based approaches for multi-omics data integration and analysis. BioData Min. 17, 38. 10.1186/s13040-024-00391-z 39358793 PMC11446004

[B4] Chakraborty C. Bhattacharya M. Lee S.-S. Wen Z.-H. Lo Y.-H. (2024). The changing scenario of drug discovery using AI to deep learning: recent advancement, success stories, collaborations, and challenges. Mol. Ther. - Nucleic Acids 35, 102295. 10.1016/j.omtn.2024.102295 39257717 PMC11386122

[B5] Chen J. Luo P. Wang C. Yang C. Bai Y. He X. (2022). Integrated single-cell transcriptomics and proteomics reveal cellular-specific responses and microenvironment remodeling in aristolochic acid nephropathy. JCI Insight 7, e157360. 10.1172/jci.insight.157360 35852860 PMC9462482

[B6] Chen C. Wang J. Pan D. Wang X. Xu Y. Yan J. (2023). Applications of multi‐omics analysis in human diseases. MedComm 4, e315. 10.1002/mco2.315 37533767 PMC10390758

[B7] Das S. Thakur S. Korenjak M. Sidorenko V. S. Chung F. F.-L. Zavadil J. (2022). Aristolochic acid-associated cancers: a public health risk in need of global action. Nat. Rev. Cancer 22, 576–591. 10.1038/s41568-022-00494-x 35854147

[B8] Dračínská H. Bárta F. Levová K. Hudecová A. Moserová M. Schmeiser H. H. (2016). Induction of cytochromes P450 1A1 and 1A2 suppresses formation of DNA adducts by carcinogenic aristolochic acid I in rats *in vivo* . Toxicology 344–346, 7–18. 10.1016/j.tox.2016.01.011 26845733 PMC4804751

[B9] Fan X. (2025). AI and natural medicines. Chin. J. Nat. Med. 23, 1281–1282. 10.1016/S1875-5364(25)60981-2 41260777

[B10] Favaro E. Bensaad K. Chong M. G. Tennant D. A. Ferguson D. J. P. Snell C. (2012). Glucose utilization via glycogen phosphorylase sustains proliferation and prevents premature senescence in cancer cells. Cell Metab. 16, 751–764. 10.1016/j.cmet.2012.10.017 23177934

[B11] Gao J. Zhang M. Chen Q. Ye K. Wu J. Wang T. (2025a). Integrating machine learning and molecular docking to decipher the molecular network of aflatoxin B1-induced hepatocellular carcinoma. Int. J. Surg. 111, 4539–4549. 10.1097/JS9.0000000000002455 40392001

[B12] Gao P. Cenatus S. Henley N. Pichette V. Mallette F. A. Barrera-Chimal J. (2025b). Inhibition of MCL-1 to eliminate senescent cells and mitigate renal fibrosis in aristolochic acid nephropathy. Cell Death Dis. 17, 56. 10.1038/s41419-025-08268-7 41298369 PMC12824375

[B13] Gao P. Cenatus S. Zhang D. Chu S. Henley N. Pichette V. (2026). Matricellular protein SMOC2 safeguards tubular integrity in acute kidney injury via integrin β3-dependent inhibition of CCND1-CDK4/6 axis. Mol. Biomed. 7, 11. 10.1186/s43556-026-00407-6 41663805 PMC12886620

[B14] Govind P. Pavethynath S. Sawabe M. Arai T. Muramatsu M. (2020). Association between rs1229984 in ADH1B and cancer prevalence in a Japanese population. Mol. Clin. Oncol. 12, 503–510. 10.3892/mco.2020.2021 32337031 PMC7179391

[B15] Ji H. Hu J. Zhang G. Song J. Zhou X. Guo D. (2021). Aristolochic acid nephropathy: a scientometric analysis of literature published from 1971 to 2019. Med. Baltim. 100, e26510. 10.1097/MD.0000000000026510 PMC8270620 34232183

[B16] Ji Q. Li H. Cai Z. Yuan X. Pu X. Huang Y. (2023). PYGL-Mediated glucose metabolism reprogramming promotes EMT phenotype and metastasis of pancreatic cancer. Int. J. Biol. Sci. 19, 1894–1909. 10.7150/ijbs.76756 37063425 PMC10092766

[B17] Komatsu M. Funakoshi T. Aki T. Unuma K. (2025). Aristolochic acid-induced DNA adduct formation triggers acute DNA damage response in rat kidney proximal tubular cells. Toxicol. Lett. 406, 1–8. 10.1016/j.toxlet.2025.02.006 39955082

[B18] Li Z. Zhang Y. Zhou Y. Wang F. Yin C. Ding L. (2021). Tanshinone IIA suppresses the progression of lung adenocarcinoma through regulating CCNA2-CDK2 complex and AURKA/PLK1 pathway. Sci. Rep. 11, 23681. 10.1038/s41598-021-03166-2 34880385 PMC8654884

[B19] Li M. Zhu G. Liu Y. Li X. Zhou Y. Li C. (2024). Integrated genomic and proteomic analyses identify PYGL as a novel experimental therapeutic target for clear cell renal cell carcinoma. Heliyon 10, e28295. 10.1016/j.heliyon.2024.e28295 38545181 PMC10966709

[B20] Lin P. Chan J. Y. Guan P. Hong J. H. Lim A. H. Ng C. C. (2024). Aristolochic acid‐related renal cell carcinoma exhibits a distinct tumor‐immune microenvironment favoring response to immune checkpoint blockade. J. Pathol. 264, 371–382. 10.1002/path.6349 39360336

[B21] Liu B. Xiao Y. Li H. Zhang A. Meng L. Feng L. (2020). Identification and verification of biomarker in clear cell renal cell carcinoma via bioinformatics and neural network model. Biomed. Res. Int. 2020, 6954793. 10.1155/2020/6954793 32626756 PMC7317307

[B22] Liu Y. Yang X. Gan J. Chen S. Xiao Z.-X. Cao Y. (2022). CB-Dock2: improved protein–ligand blind docking by integrating cavity detection, docking and homologous template fitting. Nucleic Acids Res. 50, W159–W164. 10.1093/nar/gkac394 35609983 PMC9252749

[B23] Liu C. Qiao R. He P. Chen W. Gao X. He F. (2025a). Mechanistic insights into aristolochic acid-induced hepatocellular carcinoma: a multi-dimensional analysis integrating network toxicology, machine learning, and molecular dynamics simulation. Toxicon 267, 108576. 10.1016/j.toxicon.2025.108576 40921337

[B24] Liu Z. Gao H. Li G. Yu Y. Cui M. Peng H. (2025b). Genome-wide CRISPR-based screen identifies E2F transcription factor 1 as a regulator and therapeutic target of aristolochic acid-induced nephrotoxicity. Environ. Int. 195, 109234. 10.1016/j.envint.2024.109234 39724681

[B25] Ma C. Zhang M. Tan H. Sun C. Sun S. Liu P. (2026). A Cuproptosis–Glycolysis signature predicts prognosis and highlights AURKA as a therapeutic target in ccRCC. Hum. Mutat. 2026, 6111105. 10.1155/humu/6111105 42111498 PMC13157311

[B26] Orywal K. Jelski W. Werel T. Szmitkowski M. (2018). The alterations in alcohol dehydrogenase and aldehyde dehydrogenase activities in the sera of patients with renal cell carcinoma. Adv. Med. Sci. 63, 1–4. 10.1016/j.advms.2017.05.001 28759873

[B27] Osman W. M. Youssef N. S. (2015). Combined use of COX-1 and VEGF immunohistochemistry refines the histopathologic prognosis of renal cell carcinoma. Int. J. Clin. Exp. Pathol. 8, 8165–8177. 26339385 PMC4555713

[B28] Qi J. Xing Y. Liu Y. Wang M.-M. Wei X. Sui Z. (2021). MCOLN1/TRPML1 finely controls oncogenic autophagy in cancer by mediating zinc influx. Autophagy 17, 4401–4422. 10.1080/15548627.2021.1917132 33890549 PMC8726724

[B29] Sborchia M. De Prez E. G. Antoine M.-H. Bienfait L. Indra R. Valbuena G. (2019). The impact of p53 on aristolochic acid I-induced nephrotoxicity and DNA damage *in vivo* and *in vitro* . Arch. Toxicol. 93, 3345–3366. 10.1007/s00204-019-02578-4 31602497 PMC6823306

[B30] Senkin S. Moody S. Díaz-Gay M. Abedi-Ardekani B. Cattiaux T. Ferreiro-Iglesias A. (2024). Geographic variation of mutagenic exposures in kidney cancer genomes. Nature 629, 910–918. 10.1038/s41586-024-07368-2 38693263 PMC11111402

[B31] Slade N. Moll U. M. Brdar B. Zorić A. Jelaković B. (2009). p53 mutations as fingerprints for aristolochic acid – an environmental carcinogen in endemic (balkan) nephropathy. Mutat. Res. Mol. Mech. Mutagen. 663, 1–6. 10.1016/j.mrfmmm.2009.01.005 19428366 PMC2729401

[B32] Stiborová M. Frei E. Schmeiser H. H. (2008). Biotransformation enzymes in development of renal injury and urothelial cancer caused by aristolochic acid. Kidney Int. 73, 1209–1211. 10.1038/ki.2008.125 18480852

[B33] Tran T. T. V. Surya Wibowo A. Tayara H. Chong K. T. (2023). Artificial intelligence in drug toxicity prediction: recent advances, challenges, and future perspectives. J. Chem. Inf. Model. 63, 2628–2643. 10.1021/acs.jcim.3c00200 37125780

[B34] Upadhyay R. Batuman V. (2022). Aristolochic acid I induces proximal tubule injury through ROS/HMGB1/mt DNA mediated activation of TLRs. J. Cell. Mol. Med. 26, 4277–4291. 10.1111/jcmm.17451 35765703 PMC9345294

[B35] Wang M. Wang L. Li X. Dai M. Sheng B. (2024). EDNRA regulates the tumour immune environment and predicts the efficacy and prognosis of cancer immunotherapy. J. Cell. Mol. Med. 28, e70172. 10.1111/jcmm.70172 39601333 PMC11600291

[B36] Wang C. Wang X. Deng Y. Hu Y. Hu L. (2025). Network toxicology combined with molecular docking technology to explore the molecular mechanism of amatoxin causing liver injury. Sci. Rep. 15, 26068. 10.1038/s41598-025-11720-5 40681587 PMC12274506

[B37] Wen J. Wang X. Yang G. Zheng J. (2024). AURKA promotes renal cell carcinoma progression via regulation of CCNB1 transcription. Heliyon 10, e27959. 10.1016/j.heliyon.2024.e27959 38655290 PMC11035947

[B38] Xiao L. Zhang Z. Li T. Jiang Y. Liu Y. Wang J. (2025). ETS1‐Driven nucleolar stress orchestrates OLR1^+^ macrophage crosstalk to sustain immunosuppressive microenvironment in clear cell renal cell carcinoma. Hum. Mutat. 2025, 8856239. 10.1155/humu/8856239 41080993 PMC12513783

[B39] Yang B. Xie Y. Guo M. Rosner M. H. Yang H. Ronco C. (2018). Nephrotoxicity and Chinese herbal medicine. Clin. J. Am. Soc. Nephrol. 13, 1605–1611. 10.2215/CJN.11571017 29615394 PMC6218812

[B40] Yun B. H. Sidorenko V. S. Rosenquist T. A. Dickman K. G. Grollman A. P. Turesky R. J. (2015). New approaches for biomonitoring exposure to the human carcinogen aristolochic acid. Toxicol. Res. 4, 763–776. 10.1039/C5TX00052A 26366284 PMC4564010

[B41] Zhang Q. Chen J. He H. Zhao W. Wong Y. Li W. (2024). Hepatotoxic effects of aristolochic acid: mechanisms and implications. Acta Mat. Medica 3, 349–362. 10.15212/AMM-2024-0023

[B42] Zhang B. Leung P.-C. Wong C.-K. Wang D. (2025a). Aristolochic acid I orchestrates multi-organ carcinogenesis through apoptotic pathway in bladder, kidney, and liver cancers: a multi-omics dissection. Environ. Sci. Eur. 37, 96. 10.1186/s12302-025-01146-1

[B43] Zhang X. Jin Q. Cheng G. Niu H. Yang S. Chen S. (2025b). Identification of necroptosis-associated mRNA biomarkers in kidney clear cell carcinoma. Front. Immunol. 16, 1545486. 10.3389/fimmu.2025.1545486 40969770 PMC12440986

[B44] Zhou Q. Jiang L. Su T. Liu G. Yang L. (2023). Overview of aristolochic acid nephropathy: an update. Kidney Res. Clin. Pract. 42, 579–590. 10.23876/j.krcp.22.211 37448287 PMC10565449

